# Diagnosis of autism in a rare case of tyrosine hydroxylase deficiency: a case report

**DOI:** 10.1186/s12920-023-01510-1

**Published:** 2023-04-11

**Authors:** Zoe Maria Dominique Reyes, Emma Lynch, Julia Henry, Lenika Marina De Simone, Sarah A. Sobotka

**Affiliations:** 1 Marian University College of Osteopathic Medicine, Indianapolis, IN USA; 2 Section of Developmental and Behavioral Pediatrics, Department of Pediatrics, The University of Chicago, 950 East 61St Street, Suite 207, Chicago, IL 60637 USA; 3 Section of Pediatric Neurology, Department of Pediatrics, The University of Chicago, Chicago, USA; 4 Department of Genetics, Ann and Robert H. Lurie Children’s Hospital, Chicago, USA

**Keywords:** Genetic disorders, Intellectual disability, Infantile parkinsonism, Sagawa syndrome, Developmental delay, Case report

## Abstract

**Background:**

Tyrosine hydroxylase deficiency (THD) is a rare movement disorder with broad phenotypic expression caused by bi-allelic mutations in the *TH* gene, which encode for tyrosine hydroxylase (TH) protein. Some patients with THD have improvement in dystonia with carbidopa–levodopa, a synthetic form of dopamine typically used in Parkinson’s disease, and are considered to have dopa-responsive THD. THD has been found in 0.5–1 per million persons, although due to overlapping symptoms with other disorders and the need for genetic testing, prevalence is likely underestimated. Existing literature describes some patients with THD having intellectual disability, but comorbid autism spectrum disorder (ASD) has not been reported.

**Case presentation:**

A nearly 3-year-old boy was referred to pediatric neurology due to hypotonia, delayed motor milestones, and expressive speech delay. Whole exome sequencing confirmed tyrosine hydroxylase deficiency, detecting a novel variant p.S307C first reported here. The child was treated with carbidopa–levodopa with an excellent response, resulting in improved balance, fewer falls, and improved ability to jump, run and climb stairs. He was determined to have dopa-responsive THD. Due to his delays in expressive speech, the boy also had an assessment with a developmental and behavioral pediatrician, who identified a pattern of social pragmatic speech delay, sensory sensitivities, and restricted interests, and determined that he met criteria for a diagnosis of ASD.

**Conclusions:**

While ASD can stand alone as a clinical diagnosis, it is also a cardinal feature of other genetically-based neurological disorders. To our knowledge, this is the first case that describes a patient with both disorders. Perhaps THD may be among the genetic disorders linked with ASD.

## Background

Tyrosine Hydroxylase Deficiency (THD) is a rare genetic disorder caused by bi-allelic mutations in the *TH* gene, which encode for tyrosine hydroxylase (TH) protein [1, 2]. Tyrosine hydroxylase catalyzes the conversion of l-tyrosine to l-dihydroxyphenylalanine (l-DOPA or levodopa), which is a rate-limiting step in the biosynthesis of dopamine, norepinephrine and epinephrine [3, 4]. Dopaminergic neurons are located in the substantia nigra and ventral tegmental areas of the midbrain, and projects to the basal ganglia for motor coordination, limbic system for emotion and memory formation, and the prefrontal cortex where it is involved in motor planning and cognition [5, 6]. Dopamine deficiency of any etiology can impair these processes [5–7]. THD is also known as autosomal recessive dopa-responsive dystonia. The dopa-responsive dystonias are a group of genetic syndromes caused by defects in the dopamine synthesis pathway, often associated with a therapeutic response to levodopa therapy, although some patients have lack of response or adverse responses to levodopa therapy. Three phenotypically distinct syndromes have been described based on symptoms and response to levodopa therapy. From mild to severe, they are: (1) TH-deficient dopa-responsive dystonia, (2) TH-deficient infantile parkinsonism with motor delay, and (3) TH-deficient progressive infantile encephalopathy. Mild cases may present later in childhood with lower limb dystonia and difficulty walking, worsening towards the end of the day, i.e. diurnal fluctuation [8, 9]. Dopa-responsive dystonias are estimated to have a population prevalence of 0.5–1 per million worldwide [8, 10]. Similar to other dopa-responsive dystonias, persons with THD have improvement in dystonia with carbidopa–levodopa, a synthetic form of dopamine typically used in Parkinson’s disease [8, 10]. Severe cases may present as infantile parkinsonism (hypokinesia, rigidity of extremities, tremor), with markedly delayed motor milestones and often truncal hypotonia. The most severe form, progressive infantile encephalopathy, has a much earlier onset (before 3–6 months), and may present with fetal distress, severe hypokinesia, limb hypertonia, hyperreflexia, truncal hypotonia, paroxysmal periods of lethargy alternating with irritability [11], and increased likelihood of behavioral problems and/or intellectual disabilities [8, 9]. Currently, we estimate there are over 100 reports of THD in the medical literature [12–38]. However, due to THD’s phenotypic heterogeneity and overlap with other disorders, genetic testing is necessary to confirm the diagnosis, and thus accurate estimates of population prevalence are difficult to obtain [8, 11].

In addition to these neurologic manifestations, intellectual disability has been described in cases of THD, most commonly in patients with very severe forms of THD [8, 11]. To our knowledge, there are currently no reports of autism spectrum disorder (ASD) in children with THD.

In this case report, we describe a young child presenting with delays in development, ultimately diagnosed with both THD and ASD.

## Case presentation

A nearly 3-year-old boy was referred to pediatric neurology after his Early Intervention physical therapist and his primary pediatrician found significant delays in gross motor, adaptive and social skills. Due to COVID-19, his therapies were interrupted for almost a year, and when re-initiated, his parents were most concerned about his balance; he would fall 10–20 times per day which is atypical. Overall, his development was delayed across domains: he rolled at 7 months and sat without support at 8 months. For reference, most children roll by 6 months of age and sit without support by 9 months of age [39]. Although he had first words just before a year of age, at 33 months his expressive language skills were delayed with mostly only 2-word sentences. Most children by 3 years of age are able to engage in back-and-forth conversation and answer simple questions [39]. He was also reported to have texture sensitivities, for example refusing all mushy foods. The child was born via spontaneous vaginal birth at term after an unremarkable pregnancy with Apgars of 6 and 8. He had a brief NICU course due to respiratory distress syndrome and was found to have a muscular VSD and bicuspid aortic valve. On the neurologist’s physical exam, the patient’s height, weight and head circumference were above the 99th percentile for age, per CDC standards. There were no dysmorphic features. The cranial nerve exam, including extraocular eye movements, was normal, with the exception of drooling. He had marked diffuse axial and appendicular hypotonia, without spasticity, and with normal muscle bulk. Strength was difficult to assess, however, the patient rose from the floor without assistance or Gower’s maneuver, and opened a heavy exam room door. The deep tendon reflexes were normal, with the exception of two beats of symmetrical ankle clonus. The patient energetically explored the exam room, and there was no dysmetria when reaching for objects. The gait was wide-based, with arms held in high-guard versus dystonic posture. The patient could run, but fell frequently without using his arms to brace for the fall. Family history was unremarkable for similarly affected relatives. Mother reported a history of an Individual Education Plan in school for reading and as an adult idiopathic intracranial hypertension that was treated with medication. Father reported attention deficit hyperactivity disorder but was otherwise healthy. Patient’s younger sibling was reported healthy, with normal development. There was no reported family history of neurodevelopmental disability. The maternal ancestry was Irish and German and paternal ancestry was Polish and German. Parents were non-consanguineous.

The initial diagnostic evaluation included blood tests (creatine kinase, lactate, thyroid stimulating hormone, plasma amino acids and acylcarnitine profile) and an MRI brain which were all normal (Table 1). The ongoing differential was broad, including muscle dystrophies, lysosomal storage disease, and congenital myasthenic syndromes. Given complex picture and broad differential whole exome sequencing (WES) was recommended. Meanwhile, the child’s pediatric neurologist referred him to a developmental and behavioral pediatrician to evaluate his expressive speech delay. The WES was performed at GeneDx, using buccal samples from patient and both parents. Massively parallel (NextGen) sequencing was done on an Illumina system and reads were aligned to human genome build GRCh37/UCSC hg19. The mean depth of coverage was 121×. Sequence variants are classified based on ACMG/AMP guidelines [40]. The WES revealed the patient to be compound heterozygous for two variants in *TH* gene: c.698G > A; p.R233H & c.920 C > G; p.S307C (NM_199292.2 confirming a diagnosis of autosomal recessive THD. R233H transversion is the most frequently seen pathogenic variant in individuals with THD, carried by 42 patients reported thus far in the literature [41]. The S307C variant has not previously been reported to our knowledge and is a novel variant. Authors of this publication did not conduct functional studies of the p.S307C variant. In silico analysis supports that this missense variant has a deleterious effect on protein structure/function. Provean score for this variant was − 4.83. The variant was classified as likely pathogenic. Parental testing confirmed these variants were inherited; sibling tested negative for both *TH* variants. No variants of unknown significance or variants in other candidate genes were identified on WES.

**Table 1:** Laboratory testing results

Laboratory test	Reference ranges and units	Values*
Lactic Acid	0.5–2.2 mmol/L	1.5
Vitamin D 25-OH	30.0–100.0 ng/mL	21.0*
Creatine kinase	20–200 U/L	133
TSH	0.50–6.90 u[IU]/mL	2.40
Comprehensive metabolic panel		
Glucose	60–99 mg/dL	95
BUN	5–18 mg/dL	10
Creatinine	0.31–0.47 mg/dL	0.29*
Calcium	8.8–10.8 mg/dL	9.6
Sodium	135–145 meq/L	140
Potassium	3.4–5.1 meq/L	4.0
Chloride	97–108 meq/L	105
CO_2_	20–28 meq/L	25
Anion gap	5–19 meq/L	10
Albumin	3.8–5.4 g/dL	4.4
Bilirubin, total	0–1.2 mg/dL	0.5
Alkaline phosphatase	0–280 U/L	396*
Protein, total	6–8 g/dL	6.7
ALT (SGPT)	0–41 U/L	11
AST (SGOT)	0–50 U/L	26
CBC with automated differential		
WBC	5.5–15.5 10*3/uL	8.88
RBC	3.8–5 10*6/uL	4.45
HGB	11–14 g/dL	12.7
HCT	33–42%	36.8
MCV	70–87 fL	82.7
MCH	24–31 pg	28.5
MCHC	31–37 g/dL	34.5
RDW-SD	36–51 fL	36.9
RDW-CV	11.6–14.2%	12.2
NRBCS % AUTOMATED	%	0.0
NRBCS # AUTOMATED	0–0.1 10*3 uL	< 0.01
MPV	9.2–12.8 fL	9.4
PLT	130–450 10*3/uL	262
Neutrophils #	1.5–8.5 10*3/uL	5.82
Lymphocytes #	2–9 10*3/uL	2.51
Monocyte #	0.21–1.95 10*3/uL	0.45
Eosinophils #	0–0.45 10*3 uL	0.07
Basophils #	0–0.22 10*3 uL	0.01
Immature Granulocyte #	0–0.04 10*3 uL	0.02
Neutrophils %		65.5
Lymphocytes %		28.3
Monocytes %		5.1
Eosinophils %		0.8
Basophils %		0.1
Immature granulocyte %		0.2

^*^Indicates result outside of reference range

The patient’s phenotype was considered to be mild to moderate given his age of onset, ataxia, gait, developmental delays, and lack of parkinsonian features. The patient was titrated to a final dose of carbidopa–levodopa (SINEMET) 25–100 mg tablet; ¼ tablet TID. No side effects were reported. The patient had an excellent response, resulting in improved balance, fewer falls, and improved ability to jump, run and climb stairs. These improvements confirmed that the patient had the dopa-responsive form of THD.

At 3.5 years of age, the patient presented for assessment by a developmental and behavioral pediatrician. At this time the patient had a confirmed THD diagnosis, but was not yet being treated with Carbidopa–levodopa therapy. The Developmental and Behavioral Pediatrician noted a presentation concerning for ASD: intermittent eye contact which was not sustained, difficulty with conversational reciprocity, restricted interest in letters and numbers, and sensory issues with feeding. He also had a developmental history consistent with ASD, delay in expressive language with poor social pragmatic skills (at 2 years of age he had only about 20 words which he used for labeling versus requesting or sharing). He was assessed formally with an Autism Diagnostic Observation Schedule-Module 2, which confirmed the diagnosis of ASD. The patient was referred to Applied Behavioral Analytic Therapy, a standard of care intervention for children with ASD where clinicians utilize behavior principles to (1) promote socially significant behavioral changes, (2) improve the patient’s overall adaptive behavior skills, (3) develop language and cognitive skills, and (4) decrease maladaptive behavior associated with ASD’s core symptoms. The patient was also recommended to have a chromosomal microarray and Fragile X testing, which were not performed.

## Discussion and conclusion

In this case report, we present a young boy with gross motor delays, characterized by hypotonia, dystonia and ataxia, and delays in social pragmatic speech. He was diagnosed with levodopa-responsive THD and ASD. To our knowledge, existing reports of THD have described comorbid intellectual disability but not ASD, however our case raises the possibility that THD causes or increases risk for ASD.

ASD is a complex neurodevelopmental diagnosis with clear genetic risk, based on studies of recurrence rates in siblings and twins [42–45], without a singular genetic etiology. While ASD can stand alone as a clinical diagnosis, it is also a cardinal feature of other genetically-based neurological disorders such as Fragile X Disorder (30–54% of males affected) [46–52], Tuberous Sclerosis Complex (25–50% of individuals affected) [53, 54] and Angelman Syndrome (~ 30% of individuals affected) [55]. Although ASD is a common neurodevelopmental disorder increasing in prevalence, now diagnosed in 1 in 44 children [56], it may also be possible that genetic variants in the *TH* gene may be associated with ASD. Unfortunately one of the limitations of this case is the yet unavailable information on the patient’s chromosomal microarray and Fragile X testing results, which are currently other recommended genetic tests when a child is diagnosed with ASD [57].

The prevalence of ASD has been steadily increasing over the past few decades [58–61]. One proposed cause for this increase is the greater recognition of ASD by health providers and families. Another is the growing recognition that ASD presents in a wide range of symptoms in individuals with varying levels of ability, coupled with improvements to formal diagnostic criteria systems (ICD-10; DSM-IV) [62]. This second point has led to a greater appreciation for the distinction between ASD and intellectual disability, though they are often co-occurring. Diagnostic substitution plays a role in explaining increasing ASD prevalence (and decreasing intellectual disability prevalence) [63, 64], however other factors are at play.

This patient, presenting with hypotonia, frequent falls, delays in social communication, and sensory atypicalities was found to have THD and ASD diagnoses. To our knowledge, this is the first case that describes a patient with both disorders and a novel *TH* variant (S307C). Perhaps THD may be among the genetic disorders linked with ASD and potential ASD diagnoses ought to be considered when diagnosing a child with THD who also has challenges with social communication.

## Data Availability

Data sharing is not applicable to this article as no datasets were generated or analyzed during the current study.

## Abbreviations

THD: Tyrosine hydroxylase deficiency
TH: Tyrosine hydroxylase
l-DOPA: l-Dihydroxyphenylalanine
ASD: Autism spectrum disorder
WES: Whole exome sequencing
